# Establishing an *ex vivo* porcine skin model to investigate the effects of broad-spectrum antiseptic on viable skin microbial communities

**DOI:** 10.1128/msphere.00441-25

**Published:** 2025-08-28

**Authors:** E. C. Townsend, K. Xu, K. De La Cruz, L. Huang, S. Sandstrom, J. J. Meudt, D. Shanmuganayagam, A. Huttenlocher, A. L. F. Gibson, L. R. Kalan

**Affiliations:** 1 Department of Medical Microbiology and Immunology, University of Wisconsin School of Medicine and Public Health 5232, Madison, Wisconsin, USA; 2 Microbiology Doctoral Training Program, University of Wisconsin-Madison 5228, Madison, Wisconsin, USA; 3 Medical Scientist Training Program, University of Wisconsin School of Medicine and Public Health 5232, Madison, Wisconsin, USA; 4 Department of Animal & Dairy Sciences, University of Wisconsin 5228 , Madison, Wisconsin, USA; 5 Center for Biomedical Swine Research & Innovation, University of Wisconsin School of Medicine and Public Health 5232, Madison, Wisconsin, USA; 6 Department of Pediatrics, Division of Allergy, Immunology & Rheumatology, University of Wisconsin School of Medicine and Public Health 5232, Madison, Wisconsin, USA; 7 Department of Surgery, University of Wisconsin School of Medicine and Public Health 5232, Madison, Wisconsin, USA; 8 Department of Biochemistry and Biomedical Sciences, McMaster University 3710, Hamilton, Ontario, Canada; 9 M.G. DeGroote Institute for Infectious Disease Research, McMaster University 3710, Hamilton, Ontario, Canada; 10 David Braley Centre for Antibiotic Discovery, McMaster University 3710, Hamilton, Ontario, Canada; University of Michigan Medical School, Ann Arbor, Michigan, USA

**Keywords:** viability PCR, skin microbiome, chlorhexidine gluconate, antiseptics

## Abstract

Incomplete antiseptic efficacy against potentially pathogenic microbial taxa places some patients at disproportionate risk for developing a surgical site infection. Laboratory models capable of interrogating the effects of antiseptics on the skin and its complex microbial communities are desperately needed to improve and better tailor antiseptic formulations. This work aims to establish an *ex vivo* porcine skin model to explore the impact of topical antiseptics on complex skin microbial communities and superficial skin lipids. Microbiome samples were treated with propidium monoazide to selectively evaluate DNA from viable microorganisms. Bacterial abundances were assessed via viability-qPCR and quantitative culture. Viable community populations were evaluated with 16S rRNA gene sequencing. Epidermal biopsies were collected at multiple time points for lipidomic assessment via LC/MS. The *ex vivo* environment promoted shifts in porcine skin lipid composition and microbial communities over the experiment’s duration. Compared to water-treated control skin, skin treated with the antiseptic chlorhexidine gluconate had significantly lower culturable counts and bioburden as determined by viability-qPCR. Compared to water-treated skin, viable microbial communities on CHG-treated skin displayed greater relative abundance of several gut-associated and Gram-negative bacterial taxa, including *SMB53*, *Turicibacter*, *Pseudomonas*, and *Proteus*. Collectively, these findings highlight the utility of an *ex vivo* porcine skin system for interrogating the impacts of antimicrobial disruption on complex microbial ecosystems, and ultimately, for the future testing and development of improved antiseptic formulations.

**IMPORTANCE:**

Broad-spectrum antiseptics are widely used to prevent surgical site infection and as wound cleansing agents. The impacts of such agents on beneficial microbes in the skin microbiome are understudied. Here, we describe an *ex vivo* skin model to test the impacts of antiseptics or other topical agents on the healthy skin microbiome.

## INTRODUCTION

Surgical site infections (SSI) pose a substantial burden to affected patients and the healthcare system (1–4). Pre-surgical antiseptics intentionally and effectively reduce microbial bioburden at the time of surgery to help prevent SSI from developing. However, contrary to popular belief, complete sterility is not achieved (5). Rather, antiseptics temporarily disrupt skin microbial communities and can promote enrichment of several potentially pathogenic taxa, such as *Pseudomonas*, *Escherichia*, *Acinetobacter*, and *Bacillus* (5). Incomplete antiseptic efficacy against these pathogenic Gram-negative and biofilm-forming species places some patients at disproportionate risk for developing an SSI (1, 6, 7). To better characterize the current limitations of antiseptics and ultimately improve antiseptic formulations, comprehensive laboratory models to evaluate the impacts of antiseptics on the skin and its complex microbial communities are needed.

Model systems to study skin physiology, immunology, and wound healing include animal models (e.g., mouse and porcine), reconstructed human epidermis, and *ex vivo* human or animal skin tissue (8–10). Of these, *ex vivo* porcine skin tissue may offer the greatest opportunity for interrogating the complex ecosystems of bacteria, fungi, viruses, and archaea that comprise the skin microbiome and how these communities respond to acute disruptions like antiseptic exposure. *Ex vivo* skin contains all the microstructures (e.g., hair follicles, sweat glands, and sebaceous glands) that support skin microbial communities (8). Porcine and human skin are highly similar in structure, biochemistry, keratinocyte, and resident immune cell functions (11–14), making porcine skin a valuable model for studying human skin function and wound healing (11, 14–16). Microbial communities that reside on porcine and human skin also overlap, containing 97% of the same bacterial genera (17, 18). Unlike *ex vivo* human skin tissue, which is acquired following surgical removal, porcine skin is not always treated with antiseptics prior to removal from a euthanized animal and can be acquired with its skin microbial communities intact (18).

Chlorhexidine gluconate (CHG) is one of the preferred surgical antisepsis due to its ability to reduce culturable microbial bioburden for up to 48 hours post-application (19–22). Prior to surgery, many surgical centers have patients shower with 4% CHG soap the night before and morning of their surgery. Immediately prior to incision, CHG is then applied to the surgical site (22–25). We have previously reported that CHG may have short-lived antiseptic activity but lasting cytotoxic effects on infected wound tissue (26). In addition, due to CHG’s ability to bind persistent bacterial DNA to the surface of the skin (27, 28), sequencing-based efforts to quantify and characterize the impact of CHG on the healthy skin microbiome have yielded inconsistent results (27, 29–32). To circumvent this and directly assess the impact of CHG on the viable skin microbiome of intact skin, we optimized a propidium monoazide (PMAxx)-based viability assay for selective evaluation of live microorganisms in the skin microbiome pre- and post-antiseptic exposure (5). With this method, we show that pre-operative CHG effectively reduces viable microbial bioburden but can select for potentially pathogenic taxa, particularly Gram-negative and biofilm-forming bacteria. This underscores the need to develop laboratory models for the study and improvement of antiseptic formulations. To address this, we utilized this viability assay to characterize the effects of CHG on skin microbial communities in this *ex vivo* porcine skin system.

In this work, we establish an *ex vivo* porcine skin model to explore the impact of CHG antiseptic on the skin microbiome. Consistent with our studies in human surgical populations, this work confirms that application of CHG effectively reduces viable microbial bioburden. However, this effect is temporary and accompanied by the enrichment of several potentially pathogenic taxa. We show that the laboratory model resembles a moist skin environment, and the skin undergoes lipid remodeling. Collectively, these findings highlight the advantages of an *ex vivo* porcine skin system for interrogating the impacts of topically applied antimicrobials and other chemical or mechanical disruptions to the skin microbiome.

## MATERIALS AND METHODS

### *Ex vivo* porcine skin tissue handling

*Ex vivo* porcine skin tissue was obtained from 6-month to 2-year-old Wisconsin Miniature Swine (WMS) that were bred and maintained at the University of Wisconsin–Madison. All tissues came from WMS that had been utilized and euthanized as part of other research studies being conducted that did not involve antiseptic exposure to the dorsal skin, antibiotic treatment, or induced immunocompromise. *Ex vivo* tissue was washed with sterile water and sterile gauze to remove superficial dirt and blood. Hair was removed by trimming with with scissors followed by shaving with a two- or three-blade razor. Tissue was again rinsed with sterile water and gauze until clean. Subcutaneous muscle and fat were removed, and the subcutis tissue was trimmed with a scalpel to as even a depth as possible (approximately 1 cm thick). Outer edges of the tissue, roughly 1 cm, were removed. Tissue was then divided into roughly equal, 4 × 7 inch sections, one for each experimental group. For longer experiments extending over several days, tissue sections were placed on 9 × 9-inch Dulbecco’s Modified Eagle Medium (DMEM) gel plates. These plates were made by adding 76.5% high-glucose DMEM (Cytivia, Marlborough, MA), 8.5% fetal bovine serum (FBS; Thermo Fisher Scientific, Waltham, MA), 15% of 2% agarose in 1× phosphate-buffered saline (PBS) by volume. Tissue was stored on covered DMEM gel plates at 37°C in a 5% CO_2_ incubator with daily plate changes for the duration of the experiment. Images displaying the *ex vivo* model are shown in Fig. S1.

### Maintenance of a sterile environment and antiseptic application

*Ex vivo* tissue was always handled in a biosafety cabinet that was disinfected with two rounds of 10% bleach solution followed by 70% ethanol and then UV radiation for 20 minutes before and after each use. All instruments used for tissue handling were either sterile single-use items or autoclave-sterilized reusable items. To prevent contamination from personnel, individuals handling tissue wore single-use lab coats, surgical masks, and two to three layers of gloves, with either a sterile glove outer layer or the outer layer disinfected with ethanol.

*Ex vivo* tissue was bathed with antiseptic following the instructions outlined in UW-Health pre-surgical guidelines (22), with slight modifications. In brief, the skin surface was first wet with sterile water, three pumps of 4% CHG soap (antiseptic skin cleanser; CVS Health, Woonsocket, Rhode Island) was applied and spread evenly over the surface with sterile gauze and allowed to sit for one to two minutes to allow the antiseptic to work. The antiseptic was then washed off with sterile water and patted dry with sterile gauze. To mimic local applications of CHG, 3 mL of 2% CHG in 70% isopropanol (ChloraPrep; Becton, Dickinson and Company [BD], Franklin Lakes, NJ) was applied to the skin surface, according to the manufacturer’s instructions, in a circular motion from the center of the skin surface toward the edges, and the solution was left to air dry for at least three minutes.

### Experimental design

**Fig 1:**
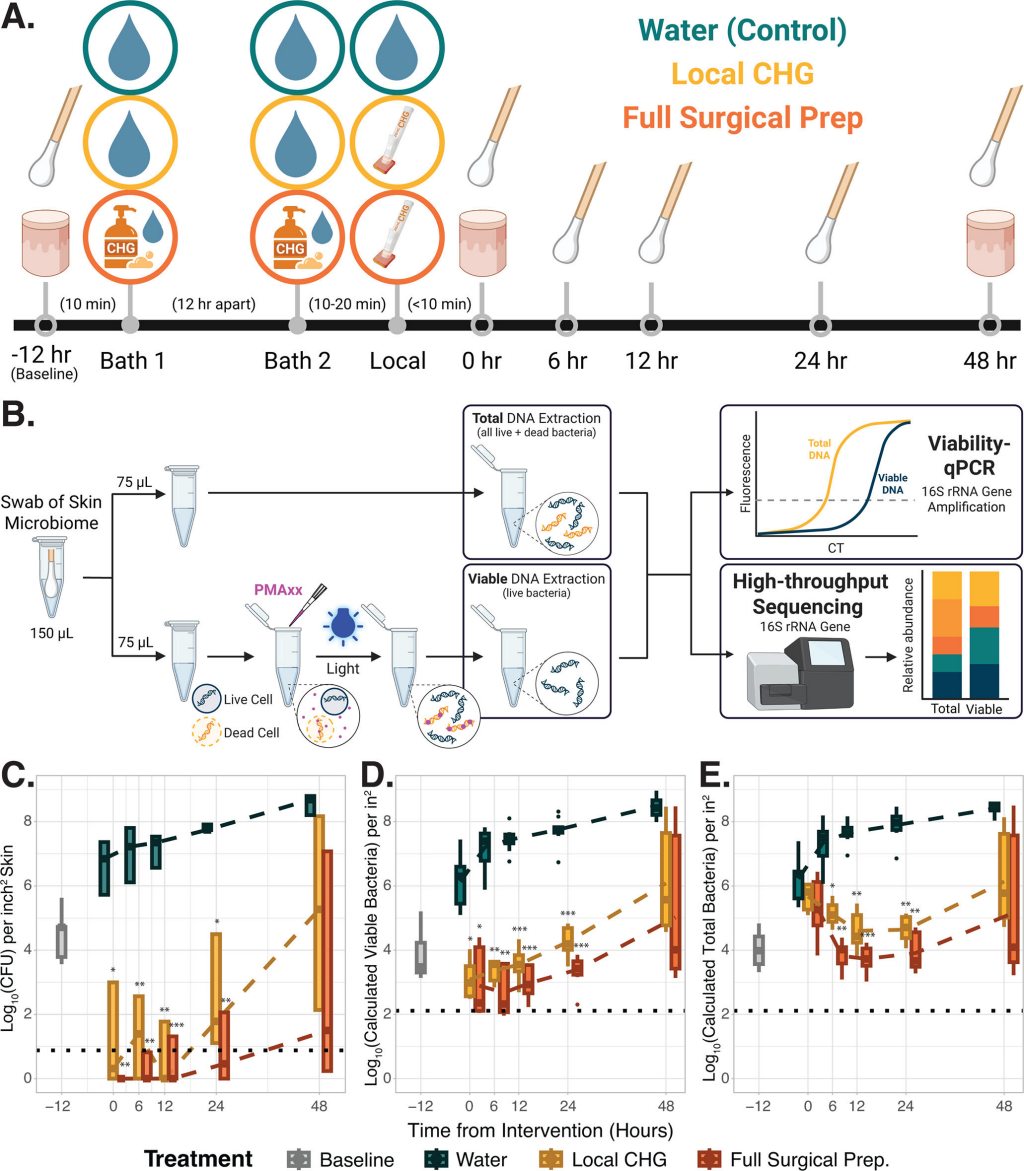
Chlorhexidine gluconate antiseptic reduces viable microbial bioburden. (**A**) Experimental design. Sections of *ex vivo* porcine skin from three porcine donors were treated as follows: (i) bathed twice with 4% CHG soap, 12 hours apart, followed by application of 2% CHG in 70% isopropanol (IPA) to mimic standard pre-surgical preparations (bottom, orange); (ii) bathed twice with sterile water, 12 hours apart, followed by a single application of 2% CHG in 70% IPA to mimic local antiseptic application (middle; yellow); or (iii) bathed three times with sterile water as a control (top; teal-green). Swabs of the skin microbiome were taken at baseline, immediately after treatment (0 hours) as well as 6, 12, 24, and 48 hours after the final treatment intervention. Microbiome samples were collected in triplicate. Punch biopsies of the epidermis from two of the porcine donors were taken at baseline, immediately after treatment intervention as well as the endpoint for lipidomic assessment. (**B**) Diagram of sample processing for viability-q-PCR and sequencing. Skin microbiome samples were split. Half of each sample was treated with PMAxx to selectively amplify DNA from viable bacteria. The remaining half of the sample remained untreated to evaluate the DNA from both live and dead bacteria (total DNA). Total and viable bioburden was evaluated via quantitative PCR of the bacterial 16S ribosomal RNA gene (viability-qPCR). Viable and total microbial community composition were then evaluated via high-throughput 16S rRNA gene sequencing. (**C**) Viable microbial bioburden per inch^2^ of skin as determined by quantitative bacterial culture for each of the experimental groups in panel **A** over time. (**D**) Viable microbial bioburden per inch^2^ of skin as determined by viability-qPCR for each of the experimental groups over time. (**E**) Total microbial bioburden per inch^2^ of skin measured by viability-qPCR for each of the experimental groups over time. (**C–E**) Bioburden in the local CHG and full surgical CHG preparation groups were compared to the water-treated control group at each respective time point via *t*-tests with Welch’s correction. For each panel, black dashed lines indicate the lower limit of each assay’s detection. *P-value < 0.05. **P-value < 0.01. ***P-value < 0.001.

To explore the effects of CHG antiseptic on the *ex vivo* porcine skin epidermal lipid composition and microbiome, sections of *ex vivo porcine* skin from three porcine donors were either (i) bathed twice with 4% CHG soap, 12 hours apart, followed by application of 2% CHG in 70% isopropanol (IPA) to mimic standard pre-surgical preparations (in orange, Fig. 1A); (ii) bathed twice with sterile water, 12 hours apart, followed by a single application of 2% CHG in 70% IPA to mimic local antiseptic application (e.g., to mimic skin preparation before a blood draw; yellow); or (iii) bathed three times with sterile water as a control (teal-green). Swabs of the skin microbiome were taken at baseline, immediately after treatment (0 hours) as well as 6, 12, 24, and 48 hours after the final treatment intervention. Microbial burden was then measured with quantitative microbial culture and viability qPCR of the bacterial 16S rRNA gene (details below). To investigate CHG’s potential effects on epidermal lipid and short-chain fatty acid profiles, punch biopsies of the epidermis were taken from two of the porcine donors in this experiment (details below).

### Sample collection

Swabs of the skin microbiome were collected from a 1-inch^2^ area of skin using the Levine technique (33). For the experiment outlined above, three samples were taken from different skin surface areas at each time point. Swabs designated for DNA extraction were placed into 155 µL of 1% bovine serum albumin (BSA) in 1× PBS and were stored at 4°C for less than 30 minutes before processing for selective detection of DNA from viable organisms (per below) and eventual DNA extraction. Swabs designated for microbial culture were also taken using Levine’s technique from a 1-inch^2^ area of the skin into 100 µL of 1% BSA in 1× PBS and were stored at 4°C for less than 2 hours before being processed for microbial culture.

### Quantitative microbial culture

Swabs designated for microbial culture were spun down using DNA IQ Spin Baskets (Promega, Madison, WI). Samples were serially diluted with 1× PBS, plated onto Tryptic Soy Agar (TSA), and then incubated at 35°C overnight for quantitative bacterial culture.

### Selective detection of DNA from viable microorganisms

Swabs collected into PBS + 1% BSA were spun down using DNA IQ Spin Baskets (Promega, Madison, WI), and each sample was split into two equal 75 µL portions. To quantify and sequence the DNA from all (live and dead) members of the *ex vivo* skin microbial communities, one portion of each sample was placed directly into −20°C storage. To selectively quantify and sequence DNA from only live microbes within skin microbial communities, the other portion of each sample was processed with a modified propidium monoazide (PMAxx, Biotium, Fremont, CA)–based viability assay (Fig. 1B). In short, PMAxx irreversibly binds to free DNA and DNA within permeable (dead) cells, allowing selective amplification and sequencing of only the non-PMAxx-bound DNA within intact (viable) cells. All steps involving PMAxx were done in a dark room. PMAxx was added to achieve a final concentration of 10 µM in each sample. Samples were rocked at room temperature for 10 minutes, exposed to blue light for 15 minutes using the PMA-Lite LED Photolysis Device (Biotium, Fremont, CA), and then spun at 5,000 × *g* for 10 minutes. Both the PMAxx-treated portion and untreated portion of each sample were stored at −20°C before DNA extraction.

Initial experiments aimed to optimize the viability assay parameters for the selective evaluation of viable microbes within the complex microbial communities residing on *ex vivo* porcine skin, both under control skin conditions and following application of CHG antiseptic. Swabs of the skin microbiome were collected from sections of *ex vivo* porcine skin. One sample served as a control to determine the anticipated amount of viable bacteria on the skin in normal circumstances. Another microbiome sample was boiled at 95°C for 10 minutes to heat-kill the majority of the bacteria present, as a negative control. A third sample was taken from a section of skin after application of 2% CHG in 70% isopropanol antiseptic. Samples were split, with half treated with PMAxx and half left untreated, and bacterial bioburden was determined by viability-qPCR of the 16S rRNA gene (Fig. 1B). The number of bacteria in the viable sample portion was divided by the number of bacteria in the total (live + dead) sample portion to determine the percentage of live bacteria within the sample. Each experimental condition was performed in *ex vivo* biological triplicates, with three technical replicates averaged for each biological sample.

### DNA/RNA extraction, DNA quantification, library construction, and sequencing

DNA extraction was performed with minor modifications, as previously described (5). Briefly, 300 µL of yeast cell lysis solution (from Epicenter MasterPure Yeast DNA Purification kit), 0.3 µL of 31,500 U/µL ReadyLyse Lysozyme solution (Epicenter, Lucigen, Middleton, WI), 5 µL of 1 mg/mL mutanolysin (M9901, Sigma-Aldrich, St. Louis, MO), and 1.5 µL of 5 mg/mL lysostaphin (L7386, Sigma-Aldrich, St. Louis, MO) were added to 150 µL of swab liquid before incubation for one hour at 37°C with shaking. Samples were transferred to 2 mL tubes containing 0.5 mm glass beads (Qiagen, Germantown, Maryland) and bead-beaten for 10 minutes at maximum speed using a Vortex-Genie 2 (Scientific Industries, Bohemia, NY), followed by a 30-minute incubation at 65°C with shaking, and a five-minute incubation on ice. The sample was spun down at 10,000 rcf for one minute, and the supernatant was added to 150 µL of protein precipitation reagent (Epicenter, Lucigen, Middleton, WI) and vortexed for 10 seconds. Samples were spun down at maximum speed (~21,000 rcf) and allowed to incubate at RT for five minutes. The resulting supernatant was mixed with 500 µL of isopropanol and applied to a column from the PureLink Genomic DNA Mini Kit (Invitrogen, Waltham, MA) for DNA purification following the recommended protocol. The concentration of extracted DNA was measured using the Qubit 4 Fluorometer (Thermo Fisher Scientific, Waltham, MA) and the accompanying protocol for dsDNA with high sensitivity. Extracted samples were stored at −20°C.

Viability quantitative polymerase chain reaction (viability-qPCR) was performed to determine the amount of DNA from viable bacteria (treated with PMAxx) and total DNA from both live and dead bacteria (non-PMAxx treated) in each sample. In short, 1 µL of extracted DNA was added to a reaction mix containing 5 µL TaqMan Fast Advanced 2X Master Mix (Applied Biosystems, Waltham, MA), 0.5 µL TaqPman 16S 20X Gene Expression Assay with FAM (Applied Biosystems), and 3.5 µL PCR pure water. Samples were run for 40 thermal cycles on the QuantStudio 7 Flex Real-Time PCR System (Applied Biosystems). Sample DNA concentrations were determined based on a standard curve of 0.015 to 15,000 pg/µL DNA extracted from *Escherichia coli* (ATCC 1496).

Samples were standardized to 2 ng/µL extracted DNA for high-throughput sequencing. 16S rRNA gene V3–V4 region amplicon libraries were constructed using a dual-indexing method at the University of Wisconsin Biotechnology Center and sequenced on a MiSeq with a 2 × 300 bp run format (Illumina, San Diego, CA). Reagent-only negative controls were carried through the DNA extraction and sequencing processes. A 20-strain staggered mix genomic material (ATCC, Manassas, VA) served as a positive sequencing control.

### Sequence analysis

The QIIME2 (34) environment was used to process DNA-based 16S rRNA gene amplicon data. Paired-end reads were trimmed, quality filtered, and merged into amplicon sequence variants (ASVs) using DADA2. Taxonomy was assigned to ASVs using a naive Bayes classifier pre-trained on full-length 16S rRNA gene 99% OTU reference sequences from the GreenGenes database (version 13_8). Using the qiime2R package, data were imported into RStudio (version 1.4.1106) running R (version 4.1.0) for further analysis using the phyloseq package (35). Negative DNA extraction and sequencing controls were evaluated based on absolute read count and ASV distribution in true samples. ASVs identified as likely experimental contaminants, unidentified reads, and reads mapped to non-bacterial taxa (e.g. chloroplasts and mitochondria) were removed from analysis. The full list of taxa IDs and sequences removed from analysis is provided on the GitHub repository. Abundances were normalized proportionally to total reads per sample. Data were imported into RStudio running R (version 4.2.1) for analysis. Plots were produced using the ggplot2 package. Taxa below 2% relative abundance were pooled into the “Other” category for the relative abundance plots. Bray-Curtis beta diversity metric was utilized to compare sample microbial community structures, and all associated plots were ordinated via non-metric multidimensional scaling (NMDS). Univariate and/or multivariate permutational multivariate analysis of variance (PERMANOVA) was used to evaluate associations between microbial community compositions and various experimental groups. Each PERMANOVA was run considering the marginal effects of terms with 9,999 permutations using Adonis2 in the vegan R package (36). Distances from a group of centroids were calculated using the vegan (36) betadisper function to evaluate the variability of microbial community compositions of samples from water-treated or CHG-treated skin. Tukey’s multiple comparisons of means was then used to determine if the degree of variability within groups was significantly different. MAASLIN2 (37) was utilized to identify significant differences in taxa abundance between various groups and significant correlations of taxa relative abundance over the time. All MAASLIN2 assessments incorporated porcine donor as a random effect.

### Lipidomics

To test for potential effects of the *ex vivo* environment and CHG exposure on the epidermal and sebaceous lipids of *ex vivo* skin, 12 mm punch biopsies were collected in triplicate from two of the three experimental replicates (pigs 2 and 3) at multiple time points (Fig. 1A). The epidermis was excised from each biopsy (Fig. S1C), placed directly into an Eppendorf tube, flash-frozen in liquid nitrogen for 10–30 seconds, and then placed immediately into a −80C freezer for storage prior to lipidomic assessment. Aliquots of the CHG solutions were also provided as controls. Lipid extraction with methyl-tert-butyl ether (38) and lipidomics were performed at the University of Wisconsin Biotechnology Center (39). Lipids were identified and quantified via LC/MS/MS and LC/MS, respectively, using both positive and negative ion modes on the Agilent 1290 Infinity II ultra-high-performance liquid chromatography (UHPLC) and the Agilent 6546 QTOF mass spectrometer (Agilent, Santa Clara, CA). Initial data processing and lipid assignment were done primarily with the Agile acquisition software (Agilent). Due to a bug in Agile software, data from the positive ion mode for pig 3 samples were processed with MS-DIAL (40). Lipid quantities in each sample were normalized by tissue mass. Results were imported into R Studio running R (version 4.2.1) for analysis. Plots were produced using the ggplot2 package. To reduce the dimensionality of the lipidomic data and explore the variability in sample lipid composition, principal coordinate analysis (PCA) was conducted using the MixOmics R package (41). Univariate and/or multivariate PERMANOVAs were used to evaluate associations between lipid compositions and various experimental groups. PERMANOVAs were all run considering the marginal effects of terms with 9,999 permutations using Adonis2 in the vegan R package (36). Differential lipid abundance between groups was assessed using DEqMS (42). Lipids were considered to be differentially abundant in a given group if they displayed log_2_(fold change) >2 and adjusted Limma *P*-value < 0.01.

### Statistical analyses

Most statistical analyses were conducted in R Studio running R (version 4.2.1). Differences between culturable, viability-qPCR–viable, and total microbial bioburden among different experimental groups were analyzed using Prism (version 9.2.0).

## RESULTS

### Selective evaluation of DNA from live skin microorganisms

To optimize viability-qPCR parameters for selective quantification of viable microbes, we determined that PMAxx concentrations of 10 µM and 25 µM yielded the most accurate quantification of live bacteria in the control *ex vivo* porcine skin sample (28 ± 7% and 18 ± 2%, respectively), compared to a heat-killed control (2 ± 2% and 3 ± 3%) or from skin treated with CHG antiseptic (10 ± 1% and 9 ± 4%; all *P*-values < 0.05 for heat-killed or CHG vs. the respective control, *t*-tests with Welch’s correction Fig. S2; Table S1). To maintain consistency with the parameters optimized for human skin microbiome samples (5), we proceeded with 10 µM PMAxx for the remaining experiments. Consistent with our observations from human skin microbiome samples (5), the comparatively low percentage of live bacteria in the control sample treated with 50 µM PMAxx (7 ± 1%) suggests that excessive PMAxx is cytotoxic.

### Chlorhexidine gluconate antiseptic reduces viable skin microbial bioburden

To explore the effects of CHG antiseptic on the *ex vivo* porcine skin microbiome, sections of *ex vivo porcine* skin from three animal donors received either (i) three CHG treatments to mimic standard pre-surgical preparations (orange, Fig. 1A); (ii) a single application of CHG in IPA to mimic local antiseptic application (yellow); or (iii) bathed three times with sterile water (teal-green). At baseline, *ex vivo* porcine skin contained roughly 10^4^ viable bacteria per square inch, as determined by quantitative microbial culture and viability PCR (Fig. 1B through D; Tables S2 and S3). Over the course of the experiment, viable microbial bioburden increased to just under 10^9^ bacteria/inch^2^ of *ex vivo* skin in the water-treated control group.

Application of CHG induced an immediate reduction in culturable bioburden (Fig. 1C). Viability-q-PCR revealed that despite negative cultures, approximately 10^3^ viable bacteria per inch squared *ex vivo* skin remained immediately following both the single CHG application and the full surgical prep (Fig. 1D). Microbial burden remained lower in the CHG treatment groups over the time course (all *P*-values < 0.05, *t*-tests with Welch’s correction, Fig. 1C and D; Table S3). Immediately after CHG application, we see no difference in total microbial bioburden (live + dead bacterial DNA) between CHG-treated groups and the water control (Fig. 1E; Table S3). This corroborates previous reports that CHG application leads to the persistence of free DNA from lysed cells on the skin. A slow decline in total DNA in the antiseptic-treated groups occurs over the first 12 hours, suggesting that this free DNA is likely slowly degraded and reused by the remaining, propagating members of the community.

### Total and viable microbial community compositions

**Fig 2:**
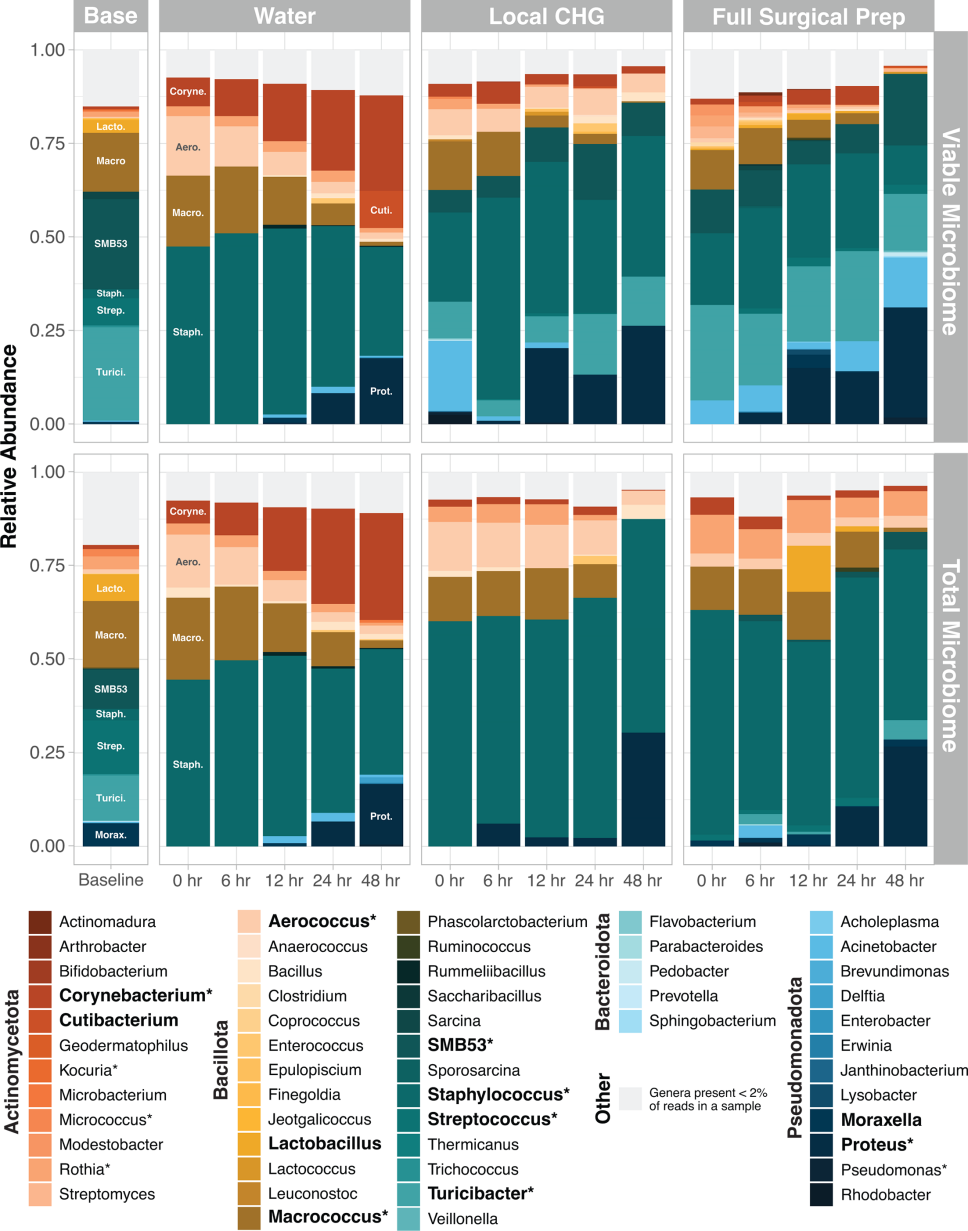
Viable and total *ex vivo* porcine skin microbial communities over time. Plots display the average relative abundance of each genus in the water (control), local CHG application, and full surgical preparation experimental groups. Average genera relative abundance within the viable and total (live + dead) microbial communities is shown in the top and bottom row, respectively. Taxa present <2% in a sample were grouped into the “Other” category. Bolded genera are those comprising at least 30% of the microbial community in at least one sample. *Genera discussed in greater detail within the text of the manuscript.

16S rRNA gene sequencing was used to characterize the impact of CHG antiseptic on viable skin microbial community structure (Fig. 1A). At baseline, *Macrococcus*, *SMB53*, *Staphylococcus*, *Streptococcus*, and *Turicibacter* were the dominant taxa within viable and total (both live + dead bacterial DNA) communities (Fig. 2). Of the genera identified, 85% have also been found on human skin (Table S4). When evaluating all samples together, porcine donor was the largest driver of microbial community composition (univariate PERMANOVA *P*-value < 0.0001, Table S5).

No significant differences were observed in viable or total microbial community structure in samples collected at baseline or from water-treated skin (Multivariate PERMANOVA *P*-values > 0.5, Fig. S3A and B; Table S6). This confirms that in normal homeostatic conditions, our viability assay effectively and selectively evaluates DNA from live microorganisms without over-representing taxa from a particular phylum or genus. Viable microbiomes are, however, significantly different than the total microbiome following CHG application in both experimental groups (all *P*-values < 0.001; Fig. S3C and D; Table S6), with total microbial communities displaying an overrepresentation of DNA from *Staphylococcus*, *Macrococcus*, *Aerococcus*, and *Rothia* (Fig. S3E through K). This supports that, following antiseptic application, free DNA from newly killed bacteria, particularly from highly abundant skin taxa, can persist on the skin and confound sequencing-based assessments if viability assays are not appropriately considered.

### Impact of the *ex vivo* environment on skin microbial communities

Changes in taxonomic proportions over time suggest that the *ex vivo* laboratory environment promotes shifts in the skin microbiome (Fig. 2; Fig. S4). Compared to baseline, water-treated skin undergoes a reduction in the relative abundance of several genera and a corresponding rise in *Staphylococcus* at the first post-treatment time point (all FDR *q*-values < 0.05 Fig. S4A through H). The two CHG-treated groups similarly displayed an increase in *Staphylococcus* relative abundance immediately post-intervention compared to baseline (all FDR *q*-values <0.1; Fig. S4E, S5A and B). Over the course of the experiment, all groups experienced significant declines in *Macrococcus* and an increase in *Proteus* (FDR *q*-value <0.05; Fig. S4I through L and S5C through F). Collectively, these findings highlight that some viable microbial community changes in the CHG-exposed groups are partially secondary to the *ex vivo* laboratory environment.

### Chlorhexidine gluconate shifts viable skin microbiome structure

**Fig 3:**
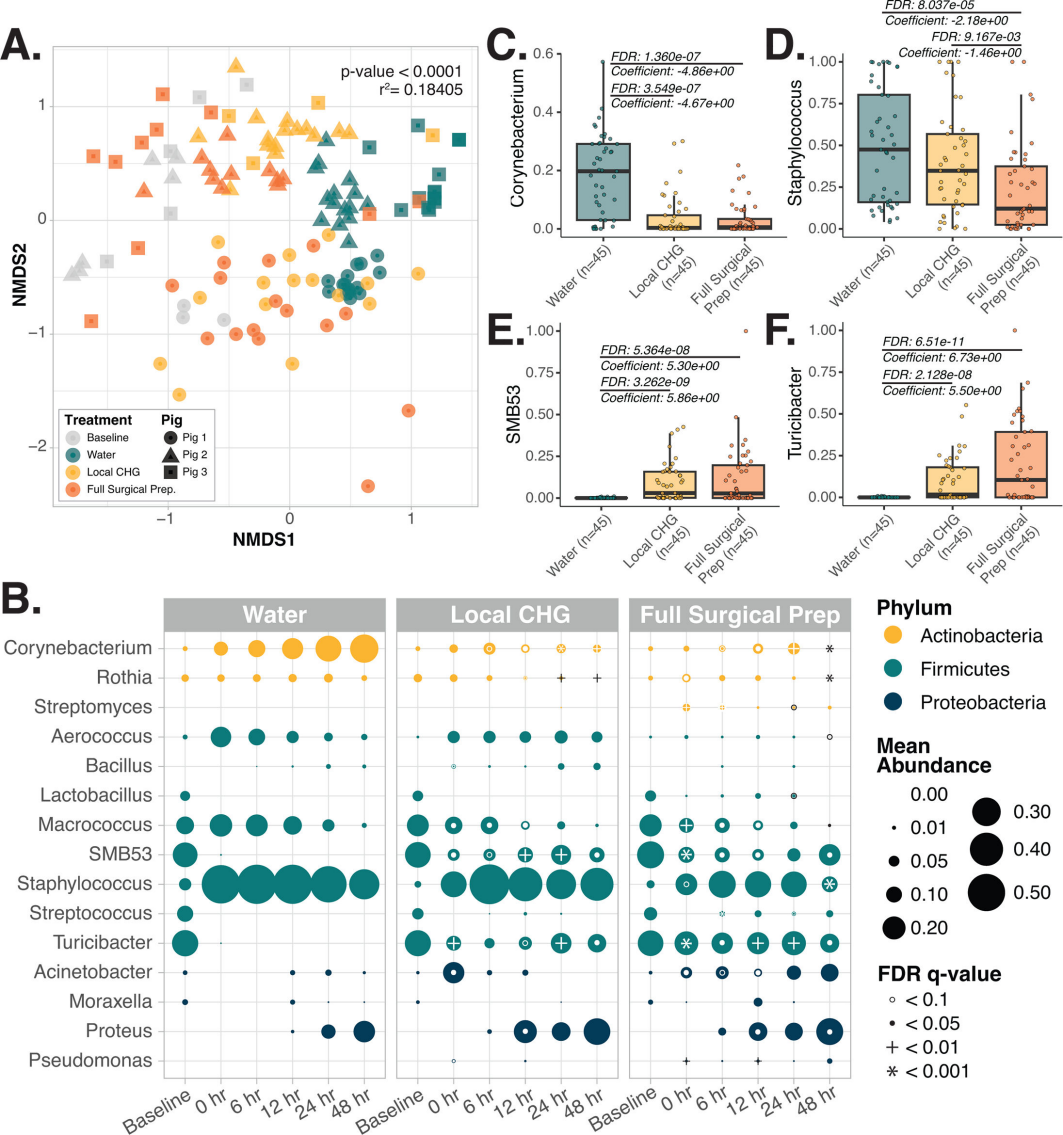
Application of CHG is associated with altered and more variable viable microbial community composition on *ex vivo* skin. (**A**) Bray-Curtis beta diversity non-metric multidimensional scaling (NMDS) ordination displaying the variability in sample viable microbial community composition in samples collected at baseline or from skin treated with water or CHG at all post-intervention time points (0–48 hours). Difference in microbial community composition between these groups was evaluated via multivariate PERMANOVA (Table S7). (**B**) Plot displaying the change in the average relative abundance of key taxa in the viable microbiome on *ex vivo* porcine skin treated with water, a single local CHG application, or the full surgical CHG antiseptic preparation over the course of the experiment. Average taxa abundance across samples is indicated by the size of the point. Differential relative abundance of taxa in the CHG treatment groups to the water treatment group at each respective time point was evaluated via MAASLIN2 accounting for the porcine donor as a random effect. White (or in a few cases, black) circles, filled-in dots, plus sign, and asterisk indicate the degree of significance. (**C–F**). Plots illustrating significant differences in the relative abundance of key taxa in either of the CHG treatment groups compared to the water control group. MAASLIN2 was used to determine differences in the relative abundance of individual taxa between CHG-treated skin compared to water-treated skin. These assessments incorporate samples from all post-intervention time points (0–48 hours). Three microbiome samples were collected from each tissue section at each time point. All MAASLIN2 analyses incorporate porcine donor as a random effect, and FDR *q*-values calculated with the Benjamini-Hochberg correction are displayed. Additional plots are in Fig. S6.

The longitudinal experimental design served to characterize the immediate and short-term effects of CHG antiseptic on *ex vivo* skin viable microbial communities. After accounting for skin donor, the viable microbiome after local CHG application, full surgical preparation, and water control groups were significantly different from one another at all timepoints (all *P*-values < 0.01, multivariate PERMANOVA; Table S7; Fig. 3A). Samples from both CHG-treated groups also displayed significantly higher variance in microbial composition over the course of the experiment, as indicated by larger average distances from their groups’ centroid compared to samples from the water control group (Fig. 3A; Fig. S6A; Table S8). Post-intervention, both CHG-treated groups were enriched for *SMB53*, *Turicibacter*, *Pseudomonas*, and *Proteus* compared to viable microbial communities from water-treated skin (all FDR *q*-value <0.05; Fig. 3B through F; Fig. S6B through F; Table S9). Skin receiving the full pre-surgical preparation had the largest reduction in *Staphylococcus*, the most dominant commensal genera on skin (all FDR *q*-value <0.0001; Fig. 3D; Fig. S6I; Table S9).

Both CHG treatment groups displayed significantly reduced abundance of commensal *Kocuria* (both FDR *q*-value <0.05 at 0 hou vs. baseline) and increased *Macrococcus* at the first time point (both FDR *q*-value <0.1 at 0 hour vs. baseline; Fig. 3B; Fig. S7 and S8). Over the course of 48 hours, skin microbiomes from both CHG treatment groups saw significant decline in *Rothia* (FDR *q*-value <0.01; Fig. 3B; Fig. S7 and S8). The proportion of *Proteus* rose in all groups; however, this rise began up to 18 hours earlier on CHG-treated skin (between 6–12 hours and 24–48 hours in the control group), suggesting that antiseptic-induced loss of commensal taxa may allow opportunistic pathogens to dominate sooner (Fig. 3B; Fig. S5E and F).

### *Ex vivo* skin undergoes lipid remodeling over time

**Fig 4:**
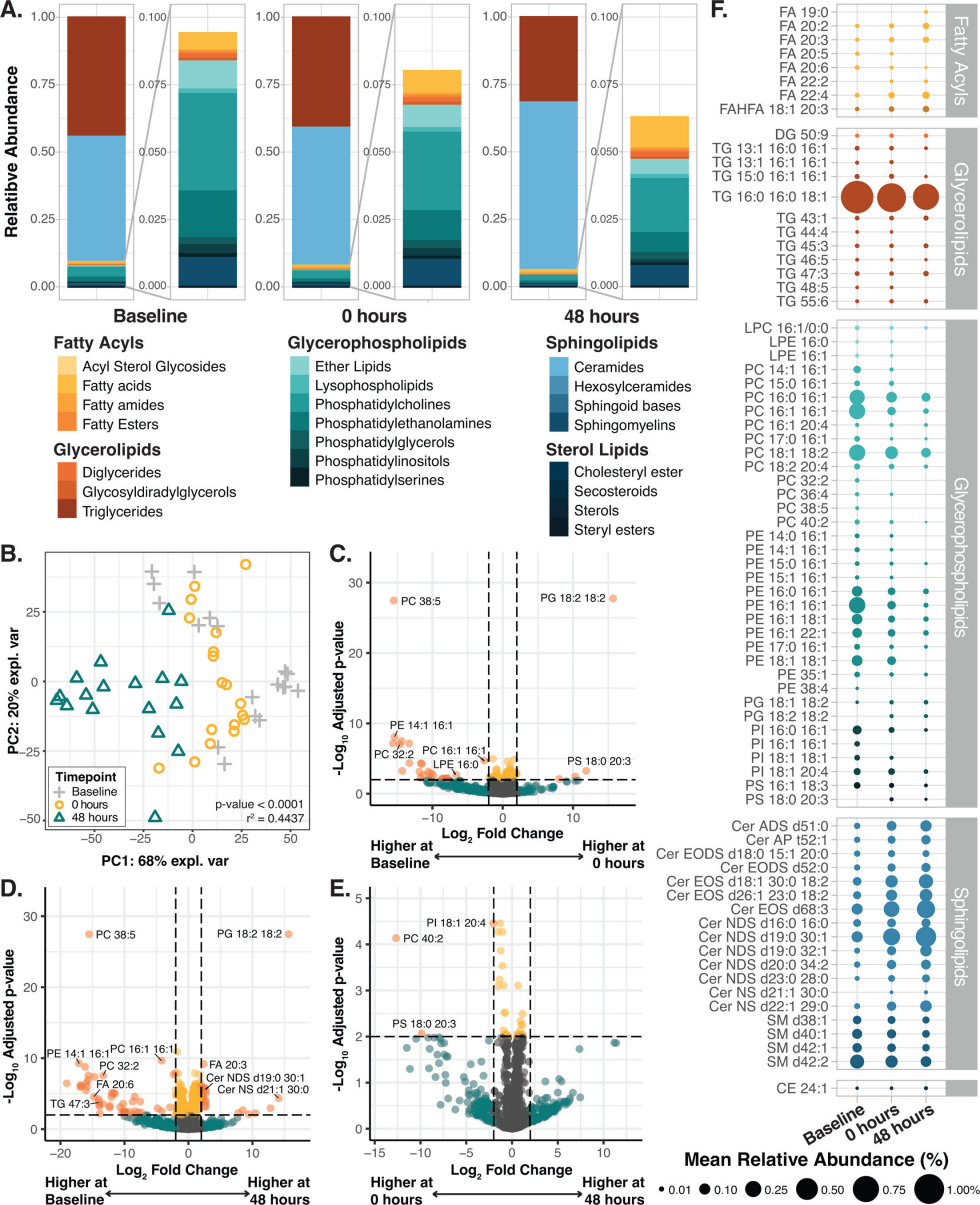
Epidermal and sebaceous lipid composition shifts over the duration of the experiment. Epidermal punch biopsies were collected at baseline as well as 0 and 48 hours following intervention for evaluation of their epidermal and sebaceous lipids (**A**) Relative abundance of lipid classes at each time point (**B**) Principal coordinate analysis (PCA) was performed to explore the variability in sample lipid composition. The plot displays the PCA ordination of samples to highlight the association between the time point of sample collection and lipid composition. Difference in lipid composition between samples collected at each time point was evaluated via univariate PERMANOVA (Table S11). Corresponding PCA plots highlighting the associations of lipid composition with porcine subject or experimental group are in Fig. S9. (**C–E**) Volcano plots displaying lipids in significantly higher or lower abundance in samples collected immediately after intervention (0 hour) vs. baseline (**C**), 48 hours after intervention vs. baseline (**D**), or 0 hours vs. 48 hours post-intervention (**E**). Differential abundance of lipids in samples from each time point was evaluated via DEqMS. Lipids were considered differentially abundant in a given group if they displayed log_2_(fold change) >2 and adjusted Limma *P*-value < 0.01. (**F**) Plot displaying the change in the average relative abundance of key lipid species on *ex vivo* porcine skin at each time point. Only lipids identified via DEqMS to be significantly more or less abundant in samples from one of the time points are included. The size of the dot indicates the average percent relative abundance. Full results are in Table S12.

Epidermal and sebaceous lipids serve as key nutrients to skin resident microbial taxa (43–45). To explore the influence of the *ex vivo* environment and CHG exposure on epidermal and sebaceous lipids, epidermal punch biopsies were collected at baseline as well as 0 and 48 hours following intervention (Fig. 1A). Mass spectrometry-based lipidomic assessment identified 1,519 lipid species across all samples (Table S10). Similar to previous lipidomic characterization of porcine and human skin (13, 46), ceramides (Cer) and triacylglycerides (TG) comprised the largest proportion of lipids on *ex vivo* porcine skin in terms of abundance (54 +/− 13% and 38 +/− 13%, respectively, for all samples; Fig. 4A; Table S10). All other groups of lipids, including fatty acids, glycerophospholipids (e.g., phosphatidylcholine), and sterol lipids, collectively comprised less than 10% of the relative abundance (Fig. 4A; Table S10). Lipid composition was not associated with either porcine subject or the treatment group, even after accounting for the time point of sample collections, suggesting that application of CHG has minimal to no impact on skin lipids (Fig. S9A and B; Table S11). Instead, overall lipid composition was significantly associated with the time point of sample collection (univariate and multivariate PERMANOVAs < 0.0001, Fig. 4B; Table S11). Over time, the proportion of ceramides increased from 47 ± 8% to 63 ± 14% (Fig. 4A; Table S10), while triacylglycerides decreased from 44 ± 8 to 31 ± 14% at baseline and 48-hour time points, respectively. The proportion of lipids from other classes declines from 9.4% to 6.3%. Baseline samples displayed significantly greater proportions of several glycerolipids and glycerophospholipids compared to both 0 and 48 hours post-intervention (all adjusted Limma *P*-values < 0.01, Fig. 4C through F; Table S12). Meanwhile, samples collected at 48 hours post-intervention had significantly higher proportions of several fatty acids (e.g., fatty acid [FA] 19:0 and FA 20:3) and ceramides (e.g., non-hydroxy-fatty acid sphingosine ceramide [Cer NS] d21:1 30:0 and omega-hydroxy-fatty acid sphingosine ceramide [Cer EOS] d18:1 30:0 18:2) compared to baseline (all adjusted Limma *P*-values < 0.01, Fig. 4C through F; Table S12). Overall, these findings support that shifts in the epidermal and sebaceous lipid composition likely occur secondary to the removal of the skin from the animal and/or the laboratory environment.

## DISCUSSION

Incomplete antiseptic efficacy against potentially pathogenic Gram-negative and biofilm-forming taxa places some patients at disproportionate risk for developing a surgical site infection (5). Laboratory models capable of interrogating the effects of antiseptics on the skin and its complex microbial communities are desperately needed to improve and better tailor antiseptic formulations. Within this work, we establish an *ex vivo* porcine skin model to study the effects of topical agents on the skin microbiome. We demonstrate that application of the antiseptic CHG results in a temporary reduction of viable microbial bioburden while promoting microbiome short-term enrichment of several potentially pathogenic, Gram-negative taxa. Collectively, this work underscores the utility of the *ex vivo* porcine skin system for interrogating chemical disruptions and subsequent recovery of the skin microbiome.

Longitudinal microbial and lipidomic assessments served to evaluate the similarities of the *ex vivo* porcine skin to that of live humans and pigs as well as the impacts of environment on the *ex vivo* tissue and microbial communities. Prior to the application of the antiseptic CHG, viable microbial communities on *ex vivo* porcine skin were dominated by taxa within the *Macrococcus*, *SMB53*, *Staphylococcus*, *Streptococcus*, and *Turicibacter* genera, consistent with skin microbiome reports from live pigs (17, 18, 47–49). Similar to prior reports, 85% of the taxa are also observed on human skin (17). In the *ex vivo* system, porcine skin treated with sterile water underwent microbial community restructuring, gradually becoming dominated by *Corynebacterium* and *Staphylococcus*. This composition mirrors human skin microbiomes observed at moist skin sites (e.g., armpit and groin) (43, 44). Moist human skin sites are characterized by high sweat gland density and tend to have warmer skin surface temperatures, around 36.5–37.5°C (50–52). It is likely that the community shifts observed over the course of the experiment were largely secondary to the relatively warm (37°C) and moist (90–95% humidity) conditions of the experimental conditions promoting the growth of the skin taxa adapted to moist environments. This underscores the potential adaptability of this model to more accurately mimic specific skin microenvironments through modifying the incubator environment parameters.

Lipidomics showed *ex vivo* porcine skin contains triglycerides, diglycerides, free fatty acids, cholesterol esters, and sterol esters—likely derived from sebum (53)—as well as epidermally derived ceramides, phospholipids, and fatty acids (46, 54). These lipid profiles reinforce the strong similarity of porcine and human skin lipid compositions (13, 14, 46, 54, 55). Increased relative abundance of ceramides and reduced phospholipids likely represent continued cornification and desquamation without the normal shedding of “dead skin” over the course of the experiment (56–58). Consistent abundance of diacylglycerides, cholesterol esters, and sterol esters may suggest continued sebaceous gland sebum production (53). Meanwhile, the gradual decline in triglycerides may reflect increased host and microbial breakdown of triglycerides into free fatty acids over the experiment (59–61). Together, these findings reinforce the underlying strengths of an *ex vivo* porcine model for the study and inference of human skin function and the potential effects of topical treatments on the skin.

Through the use of a viability assay to more accurately measure the effects of chlorhexidine gluconate on the skin microbiome (5), the value and unbiased nature of this protocol are demonstrated through the overlap of viable and total microbiomes at baseline and on water-treated skin over time. Notably, the lack of a difference between the water control and the total microbiome of the CHG-treated groups immediately after antiseptic application underscores the importance of using a viability assay. For without the viability assay, we would not have been able to accurately characterize the impacts of an antiseptic, antibiotic, or other potentially microbially toxic exposure.

By assessing only viable microbes, we demonstrate that CHG induces an immediate reduction in viable microbial bioburden. However, as observed in surgical patients (5), CHG does not completely sterilize the skin microbiome. CHG effectively targeted skin commensals (e.g., *Corynebacterium*, *Macrococcus*, and *Staphylococcus*), while several lower abundant gut-associated and potentially pathogenic taxa persisted on the skin (e.g., *SMB53*, *Turicibacter*, *Proteus*, and *Pseudomonas*) (18, 62–65). *Proteus* and *Pseudomonas* are notable biofilm-forming Gram-negative pathogens associated with SSI, with *Pseudomonas aeruginosa* being among the most common causes of surgical site infections (1, 66–68). These findings are consistent with multiple reports that CHG is less effective against Gram-negative bacteria and microbial biofilm (69–73). It is also possible that CHG does not fully penetrate deeper skin layers and hair follicles, where many skin-associated microbes reside (69–71, 74, 75). The very low proportion of *Proteus* and *Pseudomonas* (<1%) in baseline communities also emphasizes how tolerance and resistance to antiseptics provide a less competitive niche, priming the environment for expansion following depletion of the community. Collectively, these findings underscore the importance of the skin microbiome in colonization resistance. The loss of commensal taxa following antiseptic exposure may deplete a critical protective function leading to a skin microbiome unable to defend against opportunistic pathogens (43, 76–80).

We also show that reservoirs of viable microbes can persist on skin following antiseptic exposure and are sufficient to re-establish bioburden. Unlike our findings in human subjects (5), the *ex vivo* skin microbiome did not return to its baseline microbiome composition. This reflects the absence of external microbial sources, including other skin sites on the body. Instead, repopulation is limited to the microbial species tolerating the antiseptic treatment. As such, this model cannot capture the dynamics of microbiome recovery or community homeostasis post-intervention, representing a key limitation. The *ex vivo* system also lacks circulating immune cells, which may contribute to the permissive growth environment observed, evidenced by an increase in total bioburden over 48 hours post-CHG application. Another limitation is the absence of a 70% isopropanol vehicle control to assess the specific effects of CHG. However, our primary objective was to evaluate the real-world impact of CHG as commonly used in clinical settings, where it is typically delivered in an alcohol-based formulation. Despite these limitations, the *ex vivo* skin model, when paired with viability-based methods, provides a powerful tool for evaluating topical antimicrobials against complex microbial communities and in infection-relevant contexts.

In summary, *ex vivo* porcine skin tissue is a robust model for studying skin function and microbial community dynamics (11, 12, 14). Our findings reinforce the similarities of human and porcine skin and their microbiomes and highlight the model’s potential flexibility to mimic specific skin microenvironments. With this model, we demonstrate that application of CHG antiseptic temporarily reduces viable microbial bioburden, yet selects for Gram-negative and biofilm-forming taxa, which can lead to communities dominated by potential pathogens. Collectively, these findings highlight the utility of the *ex vivo* porcine skin system for pre-clinical assessment of topical agents on the skin microbiome and lipid composition and ultimately for the testing and development of improved antiseptic formulations.

## Data Availability

Sequence reads for this project can be found under NCBI BioProject PRJNA1093136. Code for analysis and generation of figures can be found on GitHub at https://github.com/Kalan-Lab/Townsend_etal_ExVivoPorcineSkinCHG.

## SUPPLEMENTAL MATERIAL

Supplemental Figures msphere.00441-25-s0001.pdf Figures S1 to S9.

Supplemental Tables msphere.00441-25-s0002.xlsx Tables S1 to S12.

ASM does not own the copyrights to Supplemental Material that may be linked to, or accessed through, an article. The authors have granted ASM a non-exclusive, world-wide license to publish the Supplemental Material files. Please contact the corresponding author directly for reuse.
