# Supplementary material for: Establishing an ex vivo porcine skin model to investigate the effects of broad-spectrum antiseptic on viable skin microbial communities
Source: mSphere. 2025 Aug 28;10(9):e00441-25. doi: 10.1128/msphere.00441-25 (PMC12482178; doi:10.1128/msphere.00441-25)
Supplement: Supplemental Figures — Figures S1 to S9. [file msphere.00441-25-s0001.pdf]

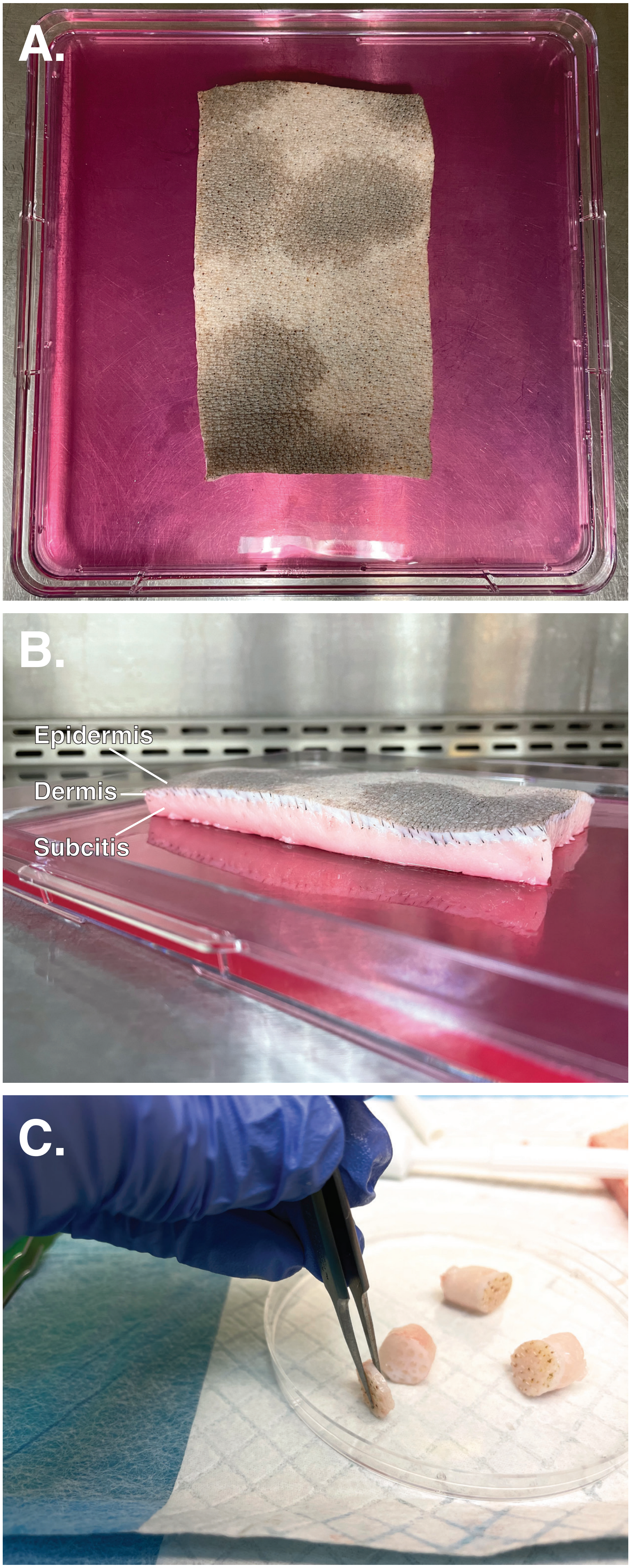

Supplemental Figure 1: The ex vivo porcine skin model. A. Photo depicting an example of the ex vivo porcine skin model. Sections of ex vivo skin were cleaned and shaved and cut into roughly 4x7 inch sections for each experimental treatment. Tissue to be used in experiments lasting more than a few hours were placed onto 9x9 inch plates of DMEM agar gel as shown. Each skin section (experimental treatment) was placed on its own separate plate. **B.** Side view of the tissue shown in panel A to highlight the epidermal, dermal, and subcutis skin layers. Note, this particular section of skin is on the thicker side of what is typically used in these experiments (about 1 cm). **C.** Photo illustrating dissection of the epidermis from 12mm punch biopsies for lipidomic assessment.

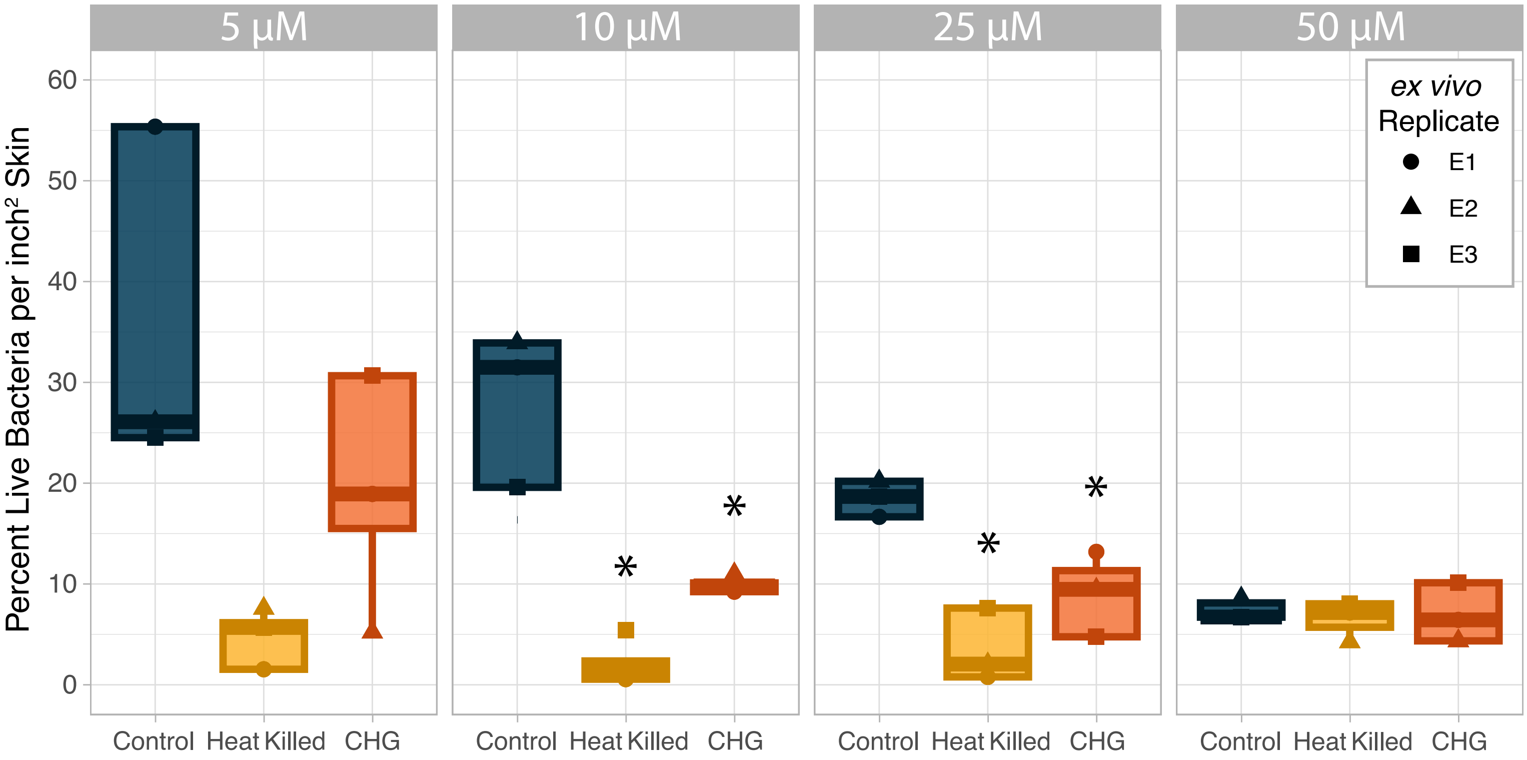

Supplemental Figure 2: Determining an optimal PMAxx concentration for selective evaluation of live bacteria within ex vivo porcine skin microbial communities. Four different concentrations of PMAxx were evaluated for accurate quantification of viable and total bacterial bioburden on ex vivo porcine skin. Swabs of the skin microbiome were collected; one sample served as a control to determine the anticipated amount of viable bacteria on the skin under normal circumstances, one microbiome sample was intentionally heat killed at 95C for 10 minutes, and one sample was taken from skin following application of CHG antiseptic (n=3 for each group). Bioburden was quantified via viability-qPCR. Percentage of live bacteria was calculated by dividing the number of viable bacteria in the sample by the total number of bacteria. The percent live bacteria in heat killed samples and samples from CHG treated skin were compared to the control group via t-tests with Welch's correction. * indicates p-value < 0.05.

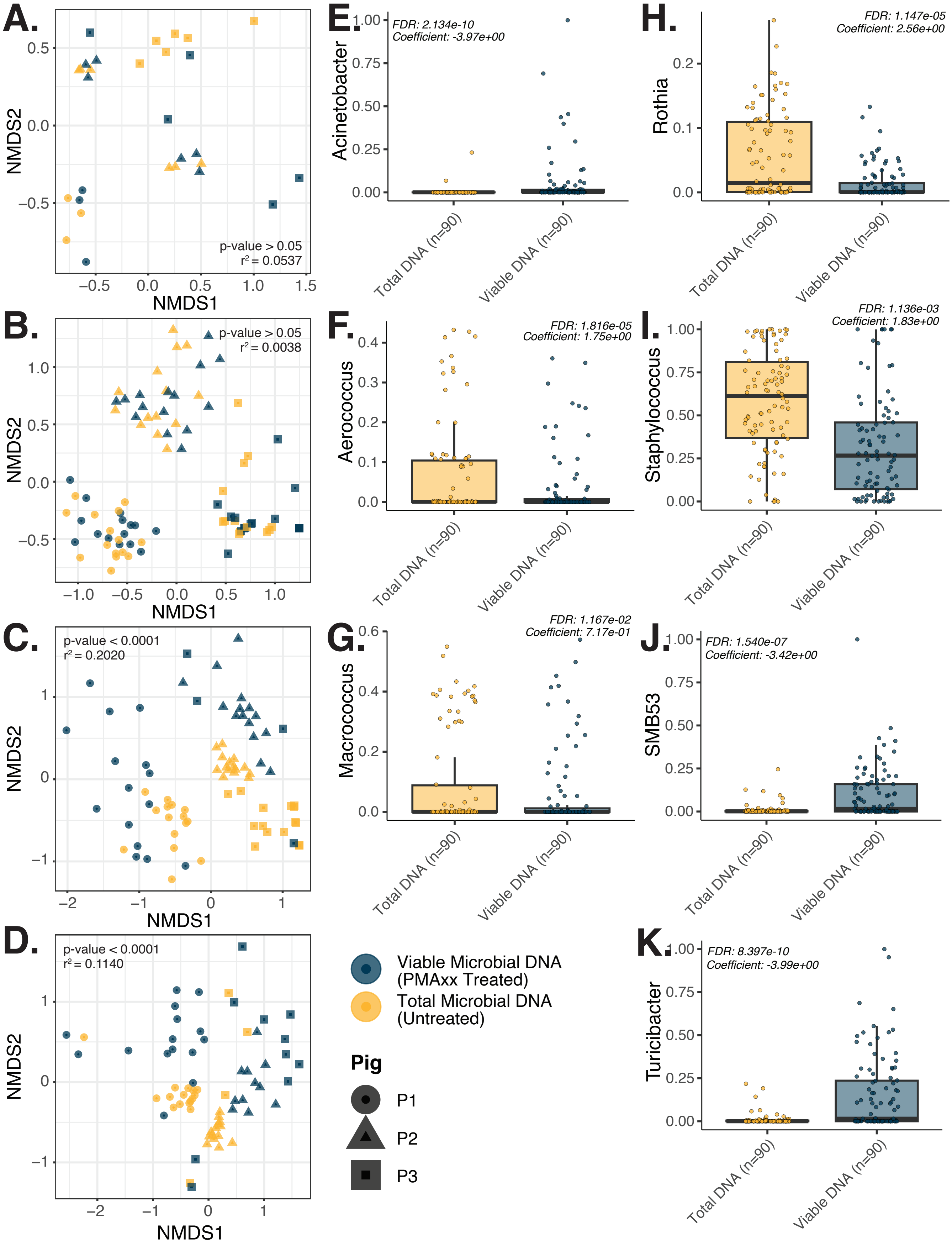

Supplemental Figure 3: Viable and total microbial community compositions differ only in the CHG treated groups after antiseptic application. A-D. Non-metric Multidimensional Scaling (NMDS) ordination of the Bray-Curtis dissimilarity matrix for samples collected at baseline **(A),** as well as in the water treated **(B),** local CHG treated **(C)**, and full-surgical CHG antiseptic preparation **(D)** groups at all post treatment timepoints (0 through 48 hours post intervention). For each panel multi-variate PERMANOVAs accounting for porcine subject with 9999 permutations were utilized to evaluate the differences between the viable (PMAxx treated; blue) and total (not treated; yellow) sample community compositions. Details can be found in supplemental table 5. **E-K.** MAASLIN2 was used to determine differences in the relative abundance of individual taxa between viable and total communities on ex vivo skin treated with CHG. Microbial communities from the local CHG and full surgical prep treatment groups at all post intervention time points (0 through 48 hours) were evaluated together. For all MAASLIN2 analyses, porcine donor was incorporated into the assessment as a random effect. FDR q-values calculated with the Benjamini-Hochberg correction are displayed.

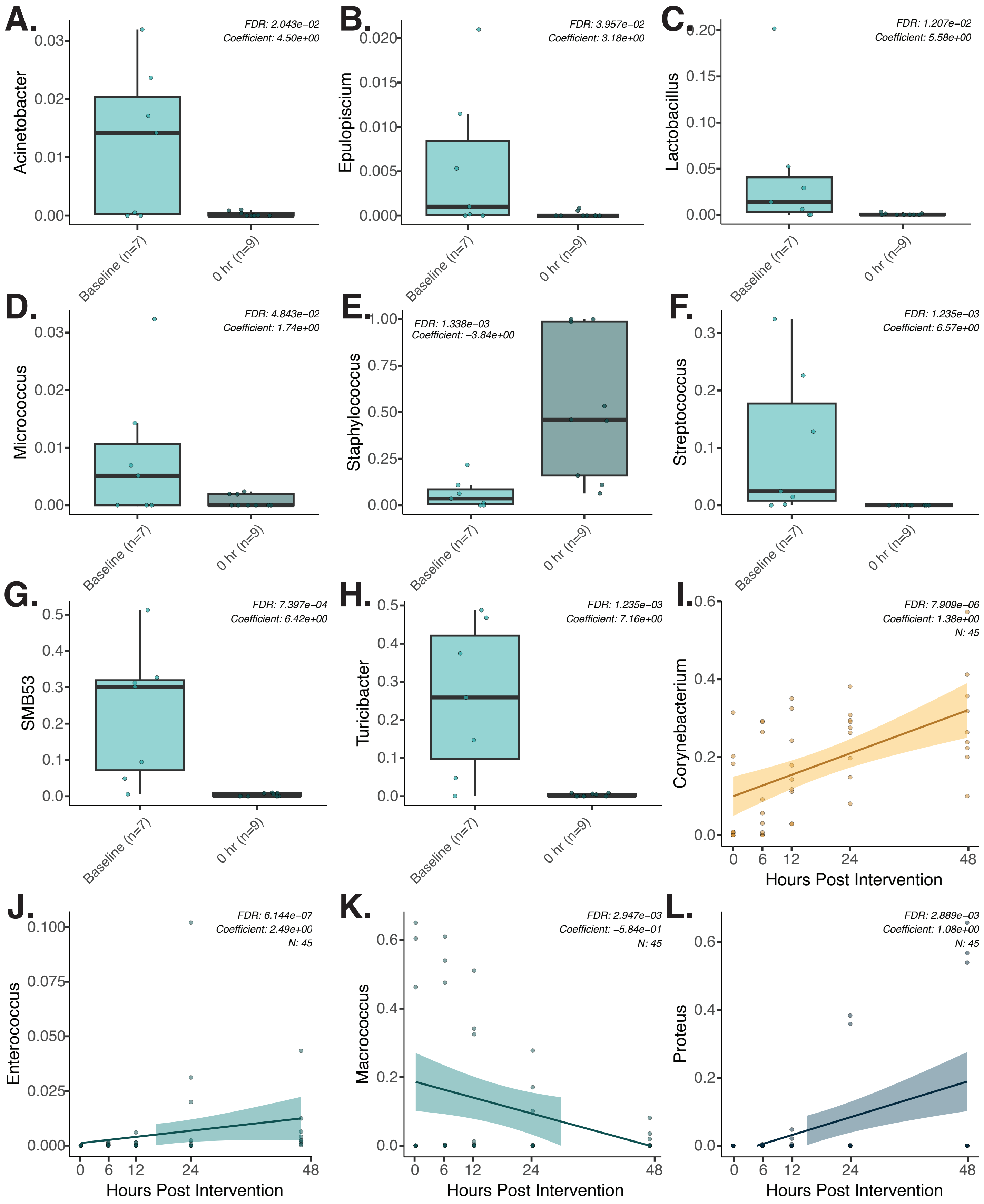

Supplemental Figure 4: The laboratory environment promotes shifts in the ex vivo porcine skin microbial communities. A-H. MAASLIN2 was used to determine differences in the relative abundance of individual taxa between the baseline and immediately post intervention (0 hr) timepoint within the viable microbial communities on control ex vivo porcine skin treated with only sterile water. FDR q-values calculated with the Benjamini-Hochberg correction are displayed. **I-L.** MAASLIN2 was also utilized to evaluate the correlations of microbial taxa relative abundance on water treated skin over time. Due to the significant community shifts that occurred between baseline and the post intervention (0hr timepoint; **A-H and Fig 3A-B**), only samples from post intervention timepoints were included in this analysis. For all MAASLIN2 analyses, porcine donor was incorporated into the assessment as a random effect. FDR q-values calculated with the Benjamini-Hochberg correction are displayed.

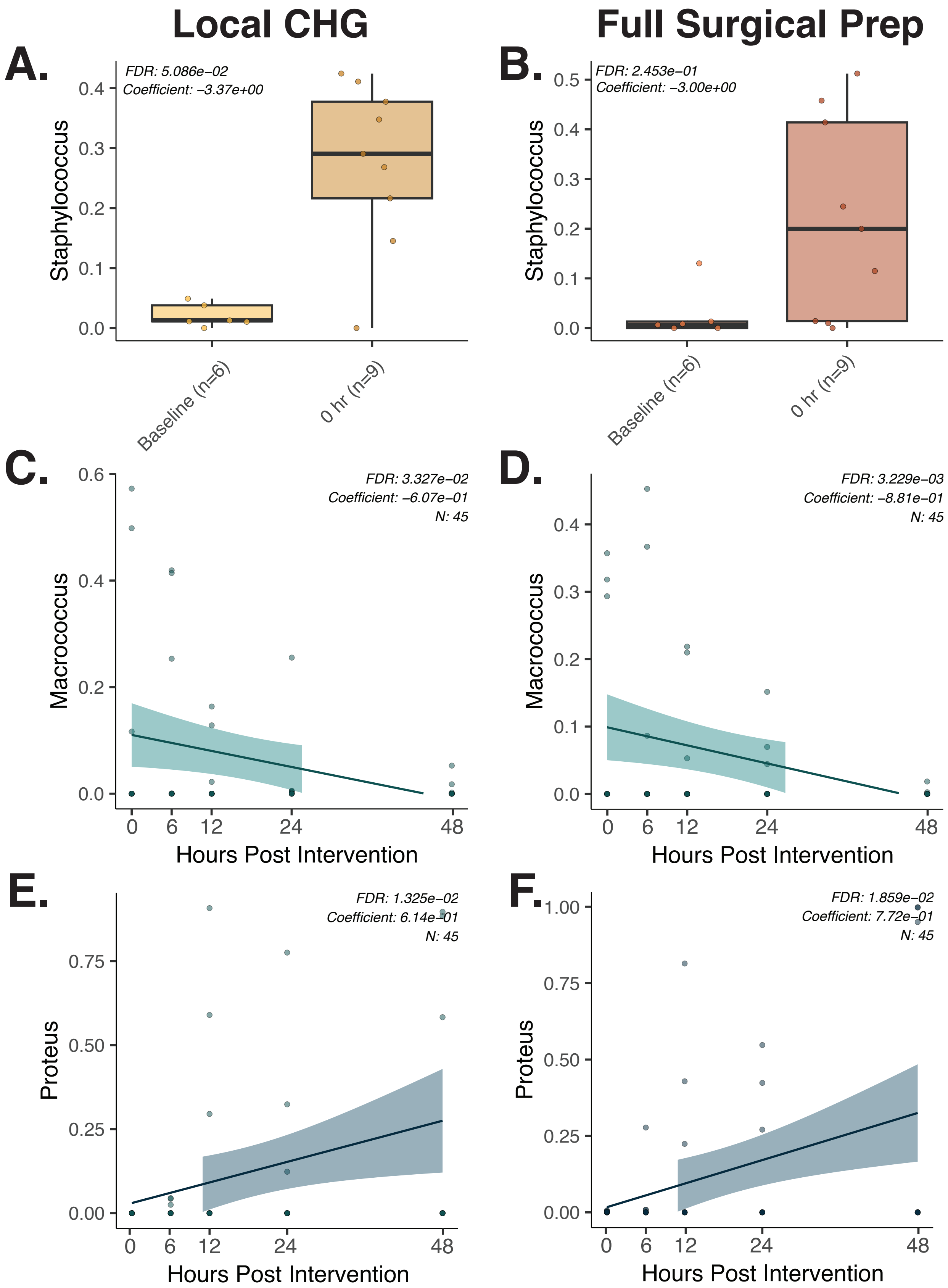

Supplemental Figure 5: Some changes seen following CHG treatment may in part be secondary to the laboratory environment. Panels highlight changes within skin microbiome following local CHG or full surgical preparation with CHG that mirror changes observed in the microbial communities on water treated skin (**Fig. S4**). Panels on the left hand side are changes seen on skin treated with local CHG and panels on the right hand side are changes seen on skin given the full surgical CHG preparation. **A-B** MAASLIN2 was used to determine differences in the relative abundance of individual taxa between the baseline and immediately post intervention (0 hr) timepoint within the viable microbial communities on ex vivo porcine skin for both of the CHG treatment groups. For all MAASLIN2 analyses, porcine donor was incorporated into the assessment as a random effect. FDR q-values calculated with the Benjamini-Hochberg correction are displayed. **C-F.** MAASLIN2 was also utilized to evaluate the correlations of microbial taxa relative abundance on CHG treated skin over time. Due to the significant community shifts that occurred between baseline and the post intervention (0hr timepoint), only samples from post intervention timepoints were included in this analysis. Additional plots displaying changes specific to either CHG treated group over time are in **Supplemental Figures 7-8**

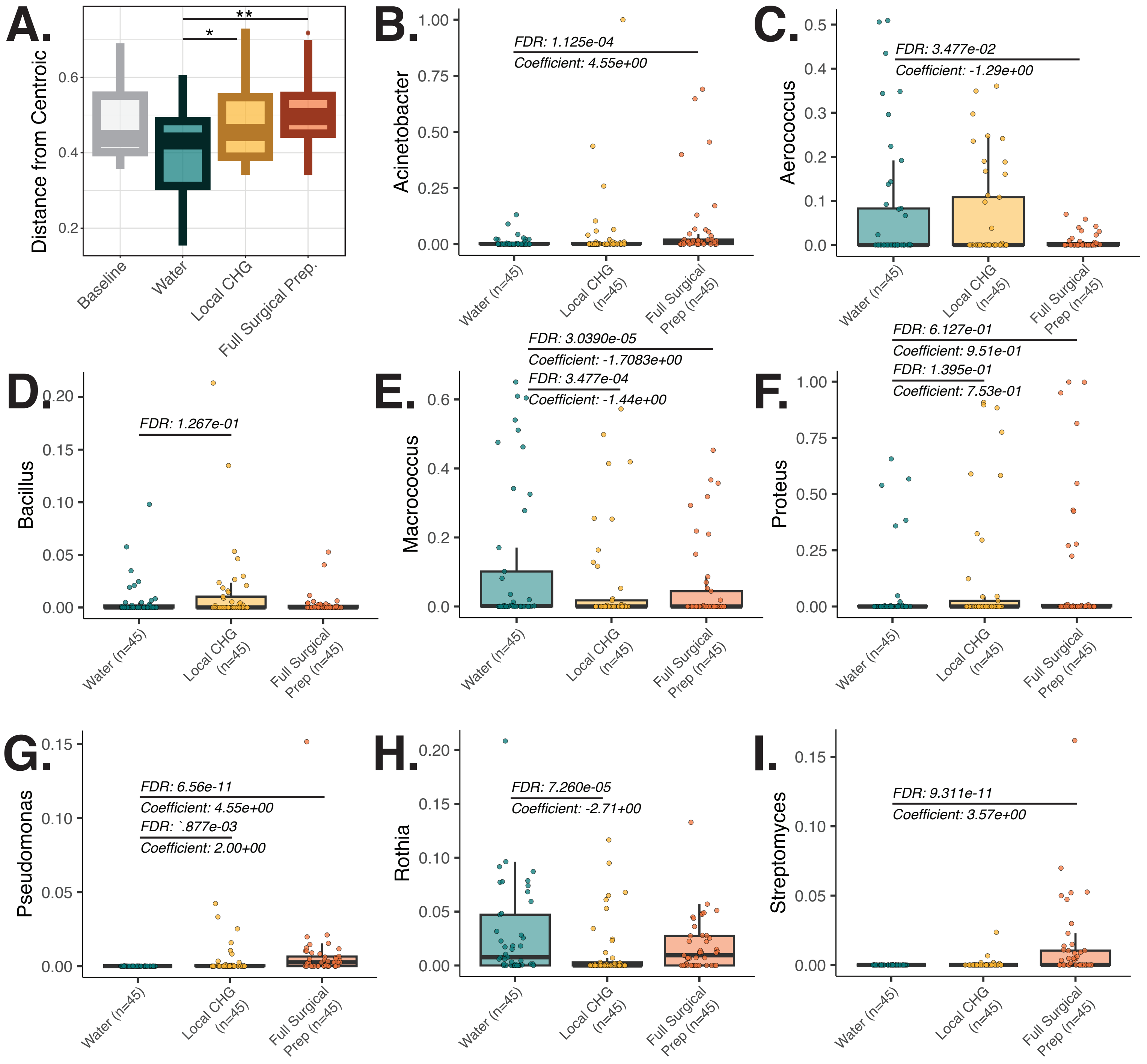

Supplemental Figure 6: Application of CHG is associated with altered and more variable viable microbial community composition. A. Companion to Figure 3A. Bray-Curtis dissimilarity metric was used to evaluate the similarity of each sample's microbial community composition. Distances from each group's centroid were calculated from this dissimilarity matrix. The water, local CHG, and full surgical prep groups include all samples collected at post intervention timepoints. Tukey multiple comparisons of means was then used to determine if the degree of variability within groups were significantly different. **B-I. Continuation of Fig 3C-F.** Relative abundance plots illustrating significant differences in key taxa in either of the CHG treatment groups compared to the water control group. MAASLIN2 was used to determine differences in the relative abundance of individual taxa between CHG treated skin compared to water treated skin. These assessments incorporate samples from all post intervention timepoints (0-48 hours). All MAASLIN2 analysis incorporate porcine donor as a random effect and FDR q-values calculated with the Benjamini-Hochberg correction are displayed. Details on significant differences between the treatment groups at each timepoint individually are displayed in **Fig 3B.**

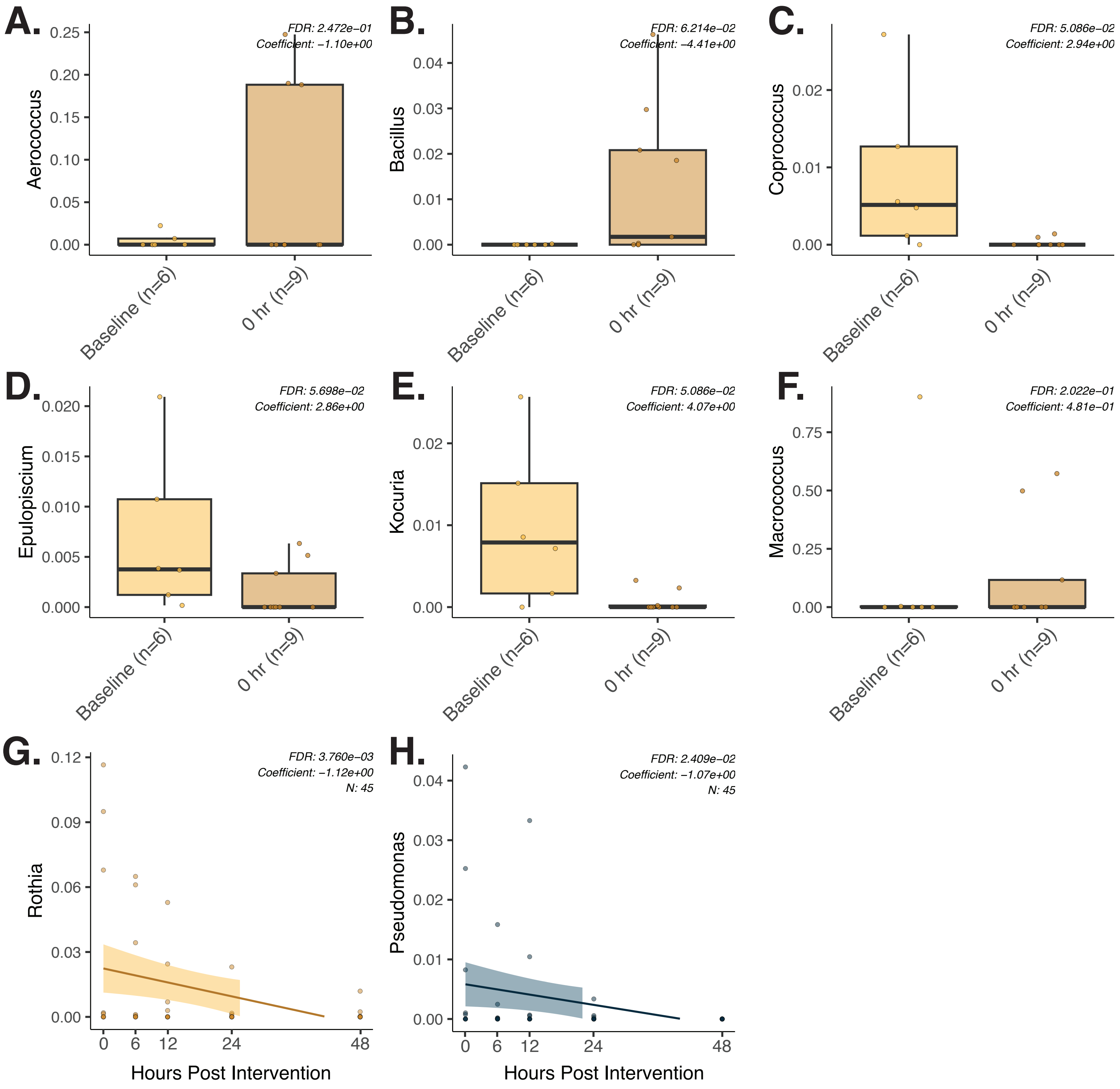

Supplemental Figure 7: Changes in microbial taxa relative abundance on skin treated with a single application of CHG. A-F. MAASLIN2 was used to determine differences in the relative abundance of individual taxa between the baseline and immediately post intervention (0 hr) timepoint within the viable microbial communities on ex vivo porcine skin treated with the single local application of CHG. For all MAASLIN2 analyses, porcine donor was incorporated into the assessment as a random effect. FDR q-values calculated with the Benjamini-Hochberg correction are displayed. **G-H**. MAASLIN2 was also utilized to evaluate the correlations of microbial taxa relative abundance on the local CHG treated skin over time. Due to the significant community shifts that occurred between baseline and the post intervention (0hr timepoint; **A-F and Fig 3B**), only samples from post intervention timepoints were included in this analysis. Additional plots displaying changes over time within the CHG treated groups that mirror those seen on water treated ex vivo skin are in **Supplemental Figure 5**.

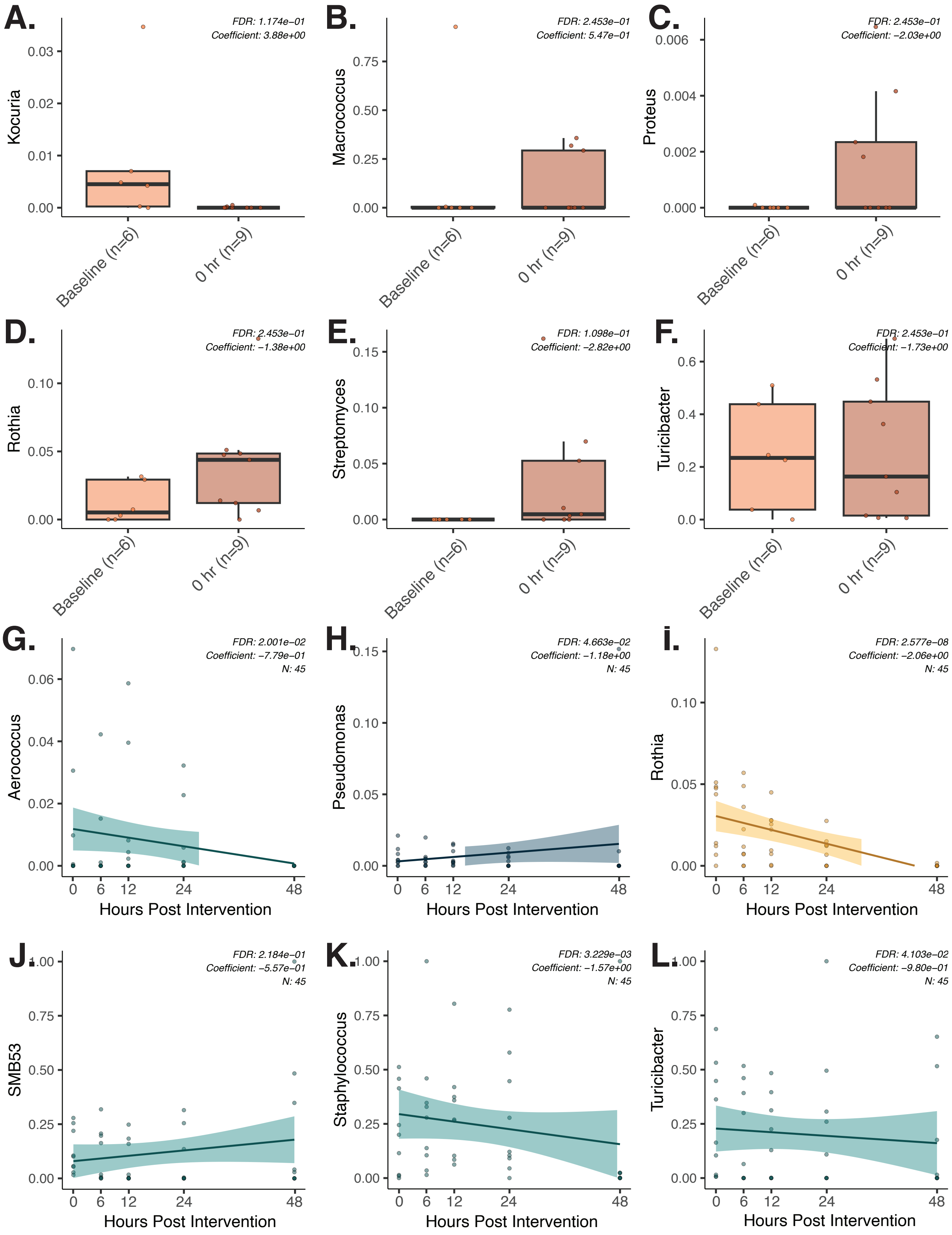

Supplemental Figure 8: Changes in microbial taxa relative abundance on ex vivo skin following the full surgical preparation with CHG. A-F. MAASLIN2 was used to determine differences in the relative abundance of individual taxa between the baseline and immediately post intervention (0 hr) timepoint within the viable microbial communities on ex vivo porcine skin treated the full surgical CHG preparation. For all MAASLIN2 analyses, porcine donor was incorporated into the assessment as a random effect. FDR q-values calculated with the Benjamini-Hochberg correction are displayed. **G-L.** MAASLIN2 was also utilized to evaluate the correlations of microbial taxa relative abundance on the full surgical prep treated skin over time. Due to the significant community shifts that occurred between baseline and the post intervention (0hr timepoint; **A-F and Fig 3B**), only samples from post intervention timepoints were included in this analysis. Additional plots displaying changes over time within the CHG treated groups that mirror those seen in on water treated ex vivo skin are in **Supplemental Figure 5**.

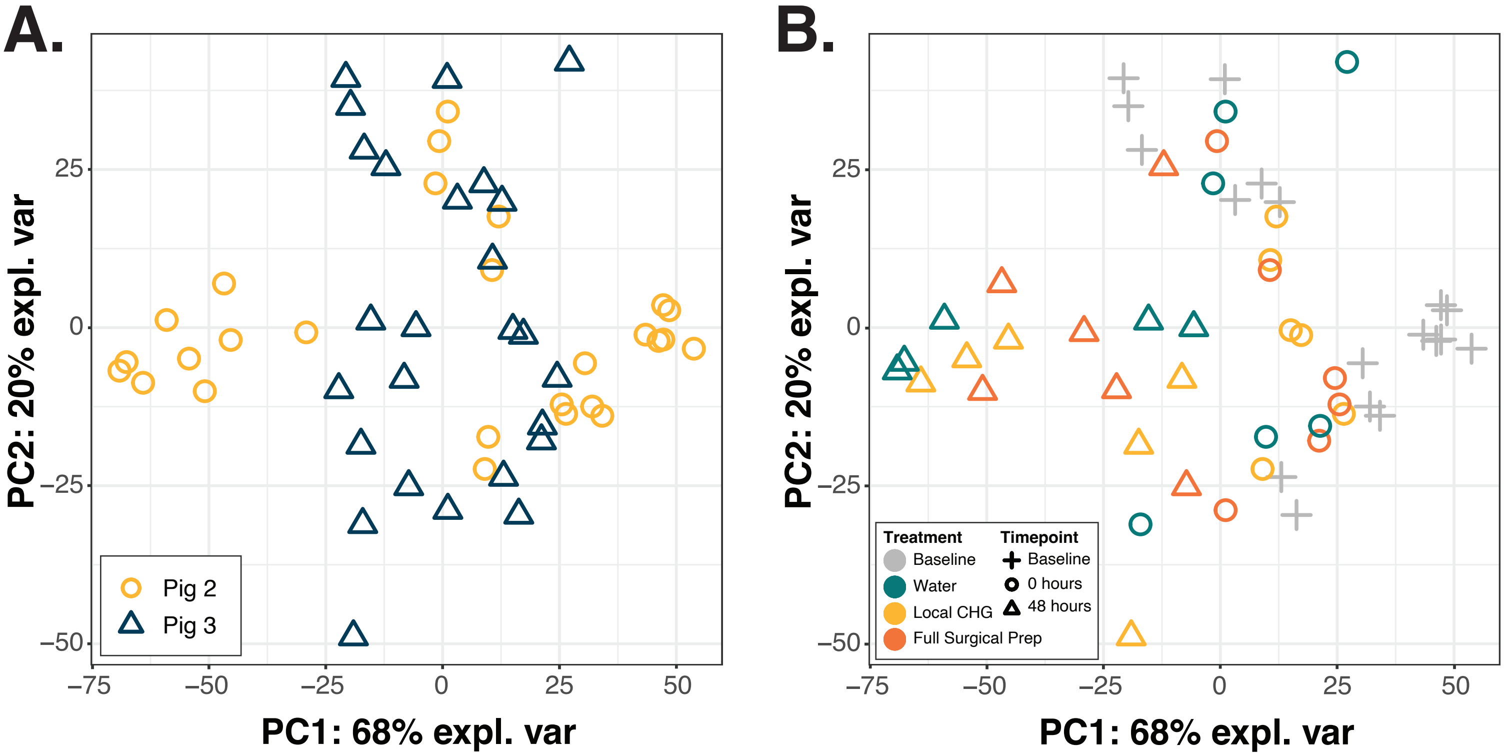

Supplemental Figure 9: Epidermal and sebaceous lipid composition is not associated with porcine subject or experimental group. A-B. Principle Coordinate Analysis (PCA) was performed to explore the variability in sample lipid composition. These plots are companion plots to **Figure 4B**. All three plots are the same ordination colored differently to highlight the (lack of) association between porcine subject **(A)** or experimental treatment group **(B)** with sample lipid composition. Difference in lipid composition between samples collected at each timepoint was evaluated via univariate PERMANOVA (both p-values > 0.1; **Table S11**).
